# Impact of a coaching program on resident perceptions of communication confidence and feedback quality

**DOI:** 10.1186/s12909-024-05383-5

**Published:** 2024-04-22

**Authors:** Carl A. Gold, Rachel Jensen, Marzena Sasnal, Heather S. Day, Rebecca K. Miller-Kuhlmann, Rebecca L. Blankenburg, Caroline E. Rassbach, Arden M. Morris, James R. Korndorffer, Aussama K. Nassar

**Affiliations:** 1 Department of Neurology & Neurological Sciences, Stanford University School of Medicine, Stanford, CA USA; 2 Department of Surgery, Stanford University School of Medicine, 300 Pasteur Drive, H3639, Stanford, CA 94305 USA; 3 Department of Surgery, Stanford-Surgery Policy Improvement Research and Education Center (S-SPIRE), Stanford University School of Medicine, Stanford, CA USA; 4 Department of Pediatrics, Stanford University School of Medicine, Stanford, CA USA

**Keywords:** Coaching, Communication skills, Resident education, Non-technical skills training, Patient experience

## Abstract

**Background:**

While communication is an essential skill for providing effective medical care, it is infrequently taught or directly assessed, limiting targeted feedback and behavior change. We sought to evaluate the impact of a multi-departmental longitudinal residency communication coaching program. We hypothesized that program implementation would result in improved confidence in residents’ communication skills and higher-quality faculty feedback.

**Methods:**

The program was implemented over a 3-year period (2019–2022) for surgery and neurology residents at a single institution. Trained faculty coaches met with assigned residents for coaching sessions. Each session included an observed clinical encounter, self-reflection, feedback, and goal setting. Eligible residents completed baseline and follow-up surveys regarding their perceptions of feedback and communication. Quantitative responses were analyzed using paired t-tests; qualitative responses were analyzed using content analysis.

**Results:**

The baseline and follow-up survey response rates were 90.0% (126/140) and 50.5% (46/91), respectively. In a paired analysis of 40 respondents, residents reported greater confidence in their ability to communicate with patients (inpatient: 3.7 vs. 4.3, *p* < 0.001; outpatient: 3.5 vs. 4.2, *p* < 0.001), self-reflect (3.3 vs. 4.3, *p* < 0.001), and set goals (3.6 vs. 4.3, *p* < 0.001), as measured on a 5-point scale. Residents also reported greater usefulness of faculty feedback (3.3 vs. 4.2, *p* = 0.001). The content analysis revealed helpful elements of the program, challenges, and opportunities for improvement. Receiving mentorship, among others, was indicated as a core program strength, whereas solving session coordination and scheduling issues, as well as lowering the coach-resident ratio, were suggested as some of the improvement areas.

**Conclusions:**

These findings suggest that direct observation of communication in clinical encounters by trained faculty coaches can facilitate long-term trainee growth across multiple core competencies. Future studies should evaluate the impact on patient outcomes and workplace-based assessments.

**Supplementary Information:**

The online version contains supplementary material available at 10.1186/s12909-024-05383-5.

## Introduction

Communication is a critical skill in graduate medical education. Resident trainees are expected to effectively communicate with patients, families, members of the health care team, and other providers. Interpersonal and communication skills are, in fact, one of the six core competencies laid out by the Accreditation Council for Graduate Medical Education (ACGME) [1]. Effective communication builds the groundwork for a strong physician–patient relationship and is associated with improved patient satisfaction, treatment adherence, and health outcomes [2–5]. While communication is an essential skill, it can be challenging to teach, directly observe, or provide constructive feedback on, especially when compared to other competencies such as patient care, medical knowledge, or technical skills.

Most of the existing programs to address the core competency of interpersonal skills and communication are episodic sessions, with substantial variability in course delivery and evaluation [6], which makes it challenging to sustain behavior change after course delivery. At our institution, we implemented the Advancing Communication Excellence at Stanford (ACES) workshop, a one-day course designed to foster relationship-centered empathetic communication [7–9]. While this was well received by residents, skill decay was an ongoing challenge due to a lack of opportunity for distributed practice to encourage skill retention and true behavior change. Additionally, true behavior change in communication requires a combination of self-reflection and targeted, high-quality feedback to serve as a catalyst for learning. Unfortunately, giving and receiving feedback continues to be a challenge in medical education; this is due to a combination of lack of direct observation at the point of care preventing objective assessment, variability in faculty expertise in providing feedback, learner differences, and time constraints [10].

To address these gaps, a longitudinal coaching program was established for residents in neurology and surgery to develop non-technical skills with a focus on communication. Non-technical skills are a set of social and cognitive skills, such as professionalism, leadership, and communication, that support the delivery of patient care within a complex system [11]. Coaching has been increasingly utilized to enhance residents’ technical and non-technical skills [12]. For instance, the Stanford Department of Pediatrics implemented a pediatrics-specific coaching program in 2014 with considerable success [13–15]. Of note, several models of coaching have been described in the medical education literature [16, 17]. In this study, coaching refers to a conceptual model of coaching encounters developed by the Stanford Pediatrics Resident Coaching Program and described in detail in a prior manuscript [13, 18].

We hypothesized that after the implementation of a longitudinal faculty-led coaching program targeting non-technical skills, surgery and neurology residents would report improved confidence in their own communication skills. We also hypothesized that residents would report higher-quality feedback from faculty after participating in the program; this is based on existing literature highlighting the importance of longitudinal relationships in feedback and the potential impact of faculty development on feedback-related behaviors [19, 20].

## Methods

### Program development

This longitudinal mixed-methods study was reviewed and deemed exempt by the Institutional Review Board at Stanford University. We designed and implemented a coaching program at Stanford University in the Departments of Neurology and Surgery in 2019. The Department of Pediatrics coaching model informed program development [13], and Kern’s 6-step approach to curriculum development was used as a conceptual framework to guide development and implementation [21].

General needs assessments in Neurology and Surgery identified communication skills training as a major opportunity (Kern’s step 1) [8, 22]. Once coaching had been identified as an intervention, we conducted a targeted needs assessment with surgery and neurology residents to identify coaching-related priorities for this key stakeholder group (Kern’s step 2). Broad goals related to learner-centered improvement in communication skills and specific objectives in terms of program design were developed, many of which are detailed in the following paragraphs (Kern’s step 3). In terms of educational strategy, several core program elements were established by the coaching program leadership team upfront, including resident-driven content, direct observation, facilitated self-reflection, targeted feedback, and goal setting (Kern’s step 4). Implementation, including stakeholder engagement and securing resources, and evaluation strategy are described in detail in a prior manuscript (Kern’s steps 5 and 6) [18].

Interested potential coaches underwent a rigorous screening process, which included completing a written application to indicate their interest and alignment of coaching with their academic goals, followed by interviews prior to selection. Coaches participated in multiple training sessions prior to working with residents in addition to ongoing monthly faculty development sessions. Each coach was assigned 8–10 resident coachees within their department. Additional details of faculty recruitment and faculty development are described in a prior manuscript [18].

Coaches were instructed to meet with their resident coachees 5–8 times over the course of the academic year for about 30–90 min per meeting. During each session, coaches were instructed to guide the resident in identifying a goal for the coaching session, directly observe a resident’s clinical encounter, facilitate the resident’s self-reflection, provide targeted feedback, and help the resident set future goals [10]. Residents were encouraged to drive the content of the sessions; for instance, they could choose to focus on specific challenges such as delivering bad news, communicating with family members, conducting a goals of care conversation, etc. Coaches did not evaluate residents’ performance.

In terms of the study setting, Stanford Health Care is a health care system that includes a 605-bed quaternary care hospital and ambulatory clinics. The hospital is a level 1 trauma center and a comprehensive stroke center. The health care system’s Patient Experience group systematically engages faculty and resident physicians through its Physician Partnership Program. Nearly all of the faculty have completed the ACES workshop, and this course has been adapted for more than 10 residency programs, including Neurology and Surgery, creating a common language around foundational communication strategies [7, 8, 23].

### Participants

All neurology and surgery residents were assigned to a faculty coach to ensure equity in the communication coaching program over a 3-year period (academic years 2019–20, 2020–21, and 2021–22). They were invited to participate in the optional research study, which included completing baseline and follow-up surveys regarding their experiences with the program.

### Data collection

We developed baseline and follow-up surveys according to best practices and a review of the literature to assess the impact of the communication coaching program on resident confidence in specific non-technical skills and perceptions of the quality of feedback received [24]. The survey was jointly developed by the research team members experienced in medical education and research to include clear and concise questions that addressed key elements of the program. Questions were asked using a 5-point scale in which participants indicated their level of agreement or confidence regarding individual statements (1 – not at all agree/confident, 2 – slightly agree/confident, 3 – somewhat agree/confident, 4 – moderately agree/confident, 5 – completely agree/confident). Surveys also included several open-ended questions to inquire about residents’ expectations and concerns about the program, helpful program elements, and opportunities for improvement (Additional File 1). The conceptual approach to the evaluation process, including survey design and piloting to ensure reliability, is described in detail in a prior manuscript [18].

All surveys were distributed via email using Qualtrics (Provo, Utah). Before the initiation of the coaching program, all residents were invited to complete the baseline survey, sent in February 2020. Residents who started in the 2020–21 and 2021–22 academic years were invited in July 2020 and June 2021, respectively. All clinically active residents who had not yet graduated were subsequently evaluated in a one-time follow-up survey sent in May 2022.

### Data analysis

We used descriptive statistics to identify participant demographics, including program and subspecialty, post-graduate year (PGY), gender identity, and prior experience with non-medical coaching, and to characterize the coach-coachee interactions during the program, including the setting and modality of feedback received from coaches (in-person, phone, etc.). We compared baseline and follow-up resident surveys using paired t-tests and defined statistical significance as a *p*-value of < 0.05. A sensitivity analysis was also performed using independent t-tests to compare responses from *all* individuals who completed the baseline survey and *all* individuals who completed the follow-up survey (SPSS software, IBM Corp, Version 28, Armonk, NY).

We performed a qualitative content analysis of open-ended item responses, a research approach to categorize and count frequencies of narrative text to identify core categories and meanings [25]. All open-ended responses were first coded inductively (codes were derived directly from the responses) in NVivo (Release 1.7.1, QSR International Pty Ltd, 2022). The codes were then quantified for core categories and counted for frequency. As the last step, the core categories were combined into two broad themes: helpful and unhelpful elements of the coaching program.

## Results

### Response rates and demographic data

**Fig. 1:**
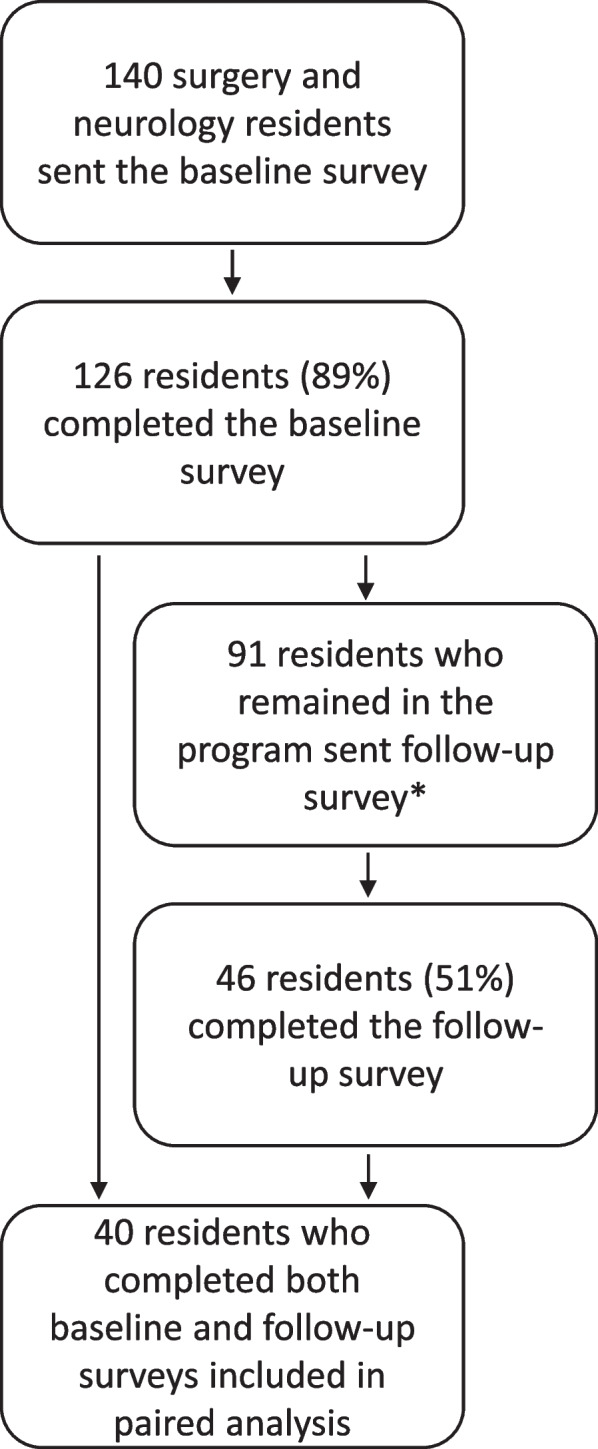
Consort diagram for survey participation. The Consort Diagram depicts survey response rates through progression of the coaching program. *51 residents were not eligible to complete the follow-up survey because they had graduated, gone on to professional development (non-clinical) time, or transitioned exclusively into programs that were not part of the coaching program

The baseline survey response rate was 90.0% (126/140). A total of 51 residents who had completed the baseline survey were not eligible to complete the follow-up survey because they had either graduated, were engaged in professional development (non-clinical) time, or had transitioned out of the department prior to the follow-up survey distribution. As such, the follow-up survey was sent to a total of 91 program participants, with a response rate of 50.5% (46/91). A total of 40 residents completed both the baseline and follow-up surveys and were included in the paired analysis. The mean time in the coaching program for residents in the paired analysis (time between the baseline and follow-up survey) was 20.1 months (SD 7.2, range: 10–28 months). See Fig. 1 for a Consort Diagram for study participation. Resident survey participant demographics are reported in Table 1.

**Table 1:** Resident (coachee) survey participant demographics

Characteristic	Baseline Survey *N* = 126 % (n)	Paired Analysis *N* = 40 % (n)
**Residency Program**		
Surgery	52.4% (66)	37.5% (15)
General	41.3% (52)	32.5% (13)
Plastics	7.1% (9)	2.5% (1)
Vascular	4.0% (5)	2.5% (1)
Neurology	47.6% (60)	62.5% (25)
Adult	35.7% (45)	50% (20)
Pediatrics	11.9% (15)	12.5% (5)
**Gender**		
Male	37.3% (47)	42.5% (17)
Female	61.1% (77)	57.5% (23)
Prefer not to state	1.6% (2)	0.0% (0)
**PGY Level**		PGY (in May/June)
PGY 1	26.2% (33)	10.0% (4)
PGY 2	31.7% (40)	22.5% (9)
PGY 3	26.1% (33)	32.5% (13)
PGY 4	11.9% (15)	25.0% (10)
PGY 5	4.0% (5)	10.0% (4)
**Prior participation in non-medical coaching**		
Yes	86.5% (109)	82.5% (33)
No	13.5% (17)	17.5% (7)
**How feedback was most often received**		
In-person		70.0% (28)
Video call		5.0% (2)
Phone		20.0% (8)
Other		5.0% (2)
**Most typical setting for observed patient encounters**		
Inpatient		50.0% (20)
Resident Continuity Clinic		30.0% (12)
Outpatient elective		7.5% (3)
Clinic Block		10.0% (4)
Other		2.5% (1)

### Baseline and follow-up coaching comparisons

#### Confidence in communication and other non-technical skills

Residents reported improved confidence in their communication skills with patients in the inpatient setting (3.7 vs. 4.3, *p* < 0.001) and clinic setting (3.5 vs. 4.2, *p* < 0.001). Residents also noted improved confidence in their communication skills with other groups, including peers (3.7 vs. 4.2, *p* = 0.001) and other members of the healthcare team (3.8 vs. 4.3, *p* = 0.002). Beyond the program’s impact on confidence in communication, residents also reported improved confidence in their skills as a resident in general (2.9 vs. 3.8, *p* < 0.001), ability to recognize their strengths and weaknesses as a physician (3.3 vs. 4.3, *p* < 0.001), and ability to set their own goals for improvement (3.6 vs. 4.3, *p* < 0.001) (Table 2).

**Table 2:** Changes in confidence in communication, self-reflection, & goal setting

Domain	Question/Statement	Baseline Survey	Follow-Up Survey	*p*-value
Communication with **patients**	*I feel confident in my communication skills:*			
	With patients in the inpatient setting	3.7	4.3	**< 0.001**
	With patients in the clinic setting	3.5	4.2	**< 0.001**
	Regarding goals of care discussions with patients and their families	3.1	4.0	**< 0.001**
Communication with **others**	*I feel confident in my communication skills:*			
	With resident peers	3.7	4.2	**0.001**
	With other members of the healthcare team	3.8	4.3	**0.002**
Self-reflection and goal setting	*I feel confident in:*			
	My skills as a resident in general	2.9	3.8	**< 0.001**
	My ability to recognize my own strengths and weaknesses as a physician	3.3	4.3	**< 0.001**
	Setting my own goals for improvement	3.6	4.3	**< 0.001**

### Resident perceptions of the quality of faculty feedback

Residents who completed both surveys reported improvements in the quality of feedback from their faculty coach compared to feedback received from faculty prior to the start of their participation in the coaching program. Statistically significant differences were observed when comparing the baseline vs. follow-up surveys for resident perceptions of the quality of feedback received on performance as a resident in general (3.2 vs. 3.8, *p* = 0.004), communication skills with patients in the inpatient setting (2.7 vs. 3.2, *p* = 0.02), communication skills with patients in the clinic setting (2.3 vs. 3.7, *p* < 0.001), and communication skills with peers (2.0 vs. 2.7, *p* = 0.02). Residents also reported improvements in the quality of feedback received from non-coach faculty (3.0 vs. 3.8, *p* = 0.001). Differences specific to the quality of feedback received were also noted; residents reported significant improvements in both the usefulness of feedback (3.3 vs. 4.2, *p* < 0.001) and whether faculty (baseline survey) or faculty coach (follow-up survey) were well-trained in providing feedback (3.1 vs. 4.3, *p* < 0.001). When receiving feedback from faculty or their faculty coach, residents also reported more opportunities to reflect on their own performance (3.6 vs. 4.6, *p* < 0.001) and set personal goals for improvement (3.0 vs. 4.3, *p* < 0.001) (Table 3). The above findings were supported by the sensitivity analysis, which compared all responses from the baseline survey with all responses from the follow-up survey (Additional File 2). This was performed as a supplemental data analysis, given the difference in sample size between the baseline and follow-up surveys.

**Table 3:** Paired comparisons of feedback received

Question/Statement	Baseline Survey	Follow-Up Survey	*p*-value
*I currently receive adequate feedback from faculty [my faculty coach] on:*			
My performance as a resident, in general	3.2	3.8	**0.004**
My communication skills with patient in the inpatient setting	2.7	3.2	**0.02**
My communication skills with patients in the clinic setting	2.3	3.7	**< 0.001**
My communication skills with my peers	2.0	2.7	**0.02**
My communication skills with other members of the health care team	2.4	2.8	0.10
My communication skills related to goals of care discussions	2.5	2.9	0.15
My professionalism skills	3.2	3.6	0.10
*I receive adequate feedback from faculty members [who are not my faculty coach]*	3.0	3.8	**0.001**
*The feedback I receive from faculty [my faculty coach] is useful*	3.3	4.2	**0.001**
*The faculty [my faculty coach] are [is] well-trained in providing feedback*	3.1	4.3	**< 0.001**
*When I receive feedback from faculty [my faculty coach] I am usually asked:*			
To reflect on my own performance	3.6	4.6	**< 0.001**
To set personal goals for improvement	3.0	4.3	**< 0.001**

### Resident perceptions of program usefulness and suggestions for improvements

In response to the open-ended questions, residents indicated helpful program elements (Table 4). Residents identified relationship-building, addressing learning needs, coaching program structure, and receiving feedback as strengths of the program. Mentorship and establishing longitudinal connections with a faculty coach were perceived as particularly valuable in residents’ professional development. The coaching program was seen as an opportunity to address specific topics, learn non-technical skills, and identify areas for improvement through timely, personalized, and structured feedback, which they described as often overlooked in medical training.

**Table 4:** Residents’ perspective of helpful program elements and areas for improvement

	**Category**	**Frequency**	**Exemplary Quotations**
**Helpful elements (*N* = 59)**	Building **relationship** with a coach / having a mentor	37% (22)	*- The mentorship is the best aspect, just getting to know this stellar faculty member on more of a personal level.* (neurology, pediatrics, PGY3) *- Having a dedicated coach has helped create a sense of community.* (surgery, general, PGY1)
	Addressing **learning needs and gaps in education**	29% (17)	*- Having observed, formal patient interviews is important as it rarely happens outside of coaching.* (neurology, adult PGY2) *- Having an objective third party observe my patient interaction and observe things that I would have completely missed.* (neurology, adult, PGY4)
	Convenient and well-designed **program structure and encounters’ settings**	19% (11)	*- I appreciated having time set aside to speak with an attending on a regular basis about my progress as a resident.* (neurology, adult, PGY3) *- [I am] glad it is taking place across different clinical settings.* (neurology, adult, PGY2)
	Receiving **feedback**	15% (9)	*- It has been wonderful to have a faculty member with whom I can meet 1-on-1 and get personalized advice/feedback and coaching.* (surgery, plastics, PGY1) *- I've received helpful and timely feedback on my communication skills, which I've been able to apply to future patient encounters.* (neurology, adult, PGY3)
**Areas for improvement (*N* = 38)**	**Logistics** difficulties	45% (17)	*- I think it is hard to coordinate meetups with faculty when you are rotating on a service they are not part of.* (surgery, plastics, PGY1) *- It can be sometimes stressful to try scheduling these sessions, especially when clinically busy.* (neurology, adult, PGY3)
	**Not addressing residents’ needs and priorities**	21% (8)	*- I do not feel like I learned a significant amount of clinically relevant skills to justify this program.* (neurology, pediatrics, PGY5) *- While there is always room for improvement in communication, at this point in my training there are more high yield topics to focus on… I feel like communication with others is pretty strong at this point and I rather just focus on other things.* (surgery, general, PGY3)
	Inflexible and artificial **structure**	24% (9)	*- Sometimes it can feel a little artificial setting up witnessed patient encounters.* (neurology, adult, PGY3) *- The framework introduced at the beginning of the year felt scripted and I'm not sure that I use it that much in my interactions with patients.* (neurology, adult, PGY3)
	**Insufficient** frequency of coaching sessions and feedback	10% (4)	*- Lack of consistent interaction with the coach.* (surgery, general, PGY4) *- [I] have not really been able to utilize the program.* (surgery, general, PGY3)

Residents also identified several program challenges and opportunities for improvement. They identified coaching session coordination as the major barrier to participation in the program due to heavy workloads or working in different locations. Scheduling sessions in advance, administrative support, incorporating sessions into clinical rotations, and meeting with the coach when at the same clinic were suggested to mitigate the logistical challenges. Some residents felt that the program did not offer enough flexibility and did not meet their needs; for example, some participants perceived that communication skills were too complex and nuanced to be learned in the “scripted” or “prescribed” way the program offered. Others felt that the discussed topics and encounter settings were not relevant to them or felt artificial. Tailoring the program to residents’ individual needs and settings, matching coaches and residents according to interest, and making the program optional for residents were described as potential actions to improve resident engagement. Additionally, lowering the coach-resident ratio was suggested to solve coaching inconsistency and infrequency issues, as well as to strengthen relationships between coaches and residents. Exemplary quotations are included in Table 4.

## Discussion

Our study evaluating the implementation of a longitudinal multi-departmental communication coaching program for residents at a large academic medical center demonstrates the feasibility and benefits on residents’ confidence in communicating with patients, peers, and others in various clinical settings and residents’ perceptions of the quality and usefulness of the feedback they receive.

The current coaching program was built around the concept of communication and, more specifically, the impact of effective communication on the patient experience [3]. While this is a historically challenging topic to teach, directly assess, or provide feedback on, this program shows that trainee-driven direct observation of communication-focused encounters can be an effective way to improve residents’ confidence in their own communication across different settings. Other programs that have implemented communication skills training have seen similar improvements in confidence [14, 26]. The fact that residents also felt more confident communicating with their peers and other healthcare team members suggests that feedback on their communication skills can be applied broadly beyond specific patient encounters. This is especially critical as the core competency of interpersonal and communication skills (ICS) applies to interactions with patients, family members, peers, and other healthcare team members both within and outside of one’s institution.

Our study also suggests that the benefits of a communication-focused coaching program extend beyond ICS and address other core competencies, such as self-reflection and goal setting, which are encompassed in the core competency of practice-based learning and improvement (PBLI) [27, 28]. The ability to think critically about one’s performance and set appropriate performance-focused individualized goals is an essential skill for physicians and helps facilitate lifelong learning. Like communication, PBLI is another historically challenging core competency for both instruction and assessment. Its sub-competencies of self-reflection and evidence-based practice are harder to observe directly and arguably even harder to teach [27, 28]. Building self-reflection and goal setting into this coaching program indirectly targets trainees’ improvement in multiple non-technical core competencies. This is imperative in the era of competency-based medical education, particularly as self-reflection and goal setting are transferrable skills that can foster self-improvement across other competencies. As residents progress in training and eventually receive patient satisfaction and performance evaluations as faculty, self-reflection, and goal setting are essential skills in implementing that feedback into their clinical practice.

Feedback, while an essential tool for self-improvement in medical training, is often lacking for trainees [19, 29, 30]. Studies have found that residents report receiving less feedback than faculty report providing [31]. Additionally, when feedback is received, it is often of inadequate quality, preventing residents from effectively integrating it for self-improvement. A qualitative analysis of fifty-one feedback-related articles found that feedback was often too lenient, too general, lacked action plans, and demonstrated clear deficiencies in delivery [30]. While there are a variety of published frameworks to support educators in how to provide effective feedback, it can be challenging to implement these resources without structured support [10, 20, 32–34]. Our results suggest that a well-designed coaching program that offers faculty development in how to provide effective feedback to trainees can improve resident perceptions of the quality of feedback received. The finding that residents perceived improved feedback after implementing the coaching program from faculty in general, not just their faculty coach, suggests an additional potential benefit of improving the *culture*of feedback more broadly. Establishing an institutional and departmental culture that normalizes high-quality feedback not only encourages teachers to prioritize their own feedback development skills but also normalizes learners' seeking and integrating feedback to assist with professional growth [35].

The study has several limitations. While our baseline survey response rate was high, the lower follow-up survey response rate limited inclusion in the paired analysis. It is plausible that disruption due to the COVID-19 pandemic, which coincided with initiation of our program, had an impact on residents’ ability or willingness to respond to surveys. The study was conducted at a single institution with funding to support faculty coach salaries, which may limit the generalizability of this program. It is possible that the time duration between baseline and follow-up surveys increased the risk of maturation bias; for instance, the authors would expect an improvement in resident perceptions of their skills as they naturally progress through training. However, the program's longitudinal design was an essential and innovative strength of the study as well.

Future areas of study include higher levels of outcomes evaluation, such as assessing for changes in resident evaluations in the core competencies of ICS and PBLI, in addition to evaluating patient satisfaction outcomes [36]. From a program development standpoint, findings from the follow-up survey are being utilized to guide programmatic improvement, including increasing the number of faculty coaches to decrease the resident-to-coach ratio.

## Conclusion

Our longitudinal resident communication coaching program has been successfully implemented over a 3-year period across two clinical departments. Based on the resident baseline and follow-up surveys, our study suggests that the program is associated with increased confidence in communication skills both with patients and other groups, increased confidence in other non-technical skills such as the ability to reflect on strengths and weaknesses and set goals, and improved perceptions of faculty feedback quality. Further research is essential as the program continues to evolve; however, the coaching program offers a critical step in supporting residents in the longitudinal development of critical non-technical skills across multiple core competencies. The next steps to build on this program implementation include expanding to other departments and developing and implementing a coaching program readiness assessment tool.

### Supplementary Information

**Supplementary Material 1. **

**Supplementary Material 2. **

## Data Availability

The study’s data are stored securely through Stanford University. The data supporting this study’s findings are not publicly available to protect participant identity. However, upon reasonable request, they are available from the corresponding author.
