# Supplementary material for: Impact of a coaching program on resident perceptions of communication confidence and feedback quality
Source: BMC Med Educ. 2024 Apr 22;24:435. doi: 10.1186/s12909-024-05383-5 (PMC11036561; doi:10.1186/s12909-024-05383-5)
Supplement: Supplementary file 1 — Supplementary Material 1. [file 12909_2024_5383_MOESM1_ESM.docx]

**Additional File 1:**

Survey Instruments

Baseline Survey

Please complete this pre-coaching assessment by DATE. This survey will take approximately 5-10 minutes and will help us improve the Coaching Initiative for other residents.

Of note, you will be asked to provide your street and 2 last digits of your cell phone as personal identifiers – it will be used to link the data from repeated outcome measures. Your identity will remain anonymous to the Coaching Initiative Steering Group.

All information obtained in this study is strictly confidential unless disclosure is required by law. We will not release information that could identify you, and when the results of this study are presented at a professional conference or published in a scholarly journal, the results will only be reported in aggregate, and your identity will not be divulged. All computerized data collected will be secured and password protected.

Thank you from the Coaching Initiative Steering Group.

**Q1 Please answer the following questions to de-identify your survey responses:**

- Street you lived on as a child
- Last 2 digits of cell phone

**Q2 I am a:**

- PGY2 adult neurology resident
- PGY3 adult neurology resident
- PGY4 adult neurology resident
- PGY5 child neurology resident
- PGY3 child neurology resident
- PGY4 child neurology resident
- PGY1 general surgery resident
- PGY2 general surgery resident
- PGY3 general surgery resident
- PGY4 general surgery resident
- PGY5 general surgery resident
- PGY1 vascular surgery resident
- PGY2 vascular surgery resident
- PGY1 plastics surgery resident
- PGY2 plastics surgery resident

**Q3 Have you ever been coached in a non-medical setting (such as music or sports) in the past? (check all that apply)**

▢ Yes - in the past 5 years

▢ Yes - more than 5 years ago

▢ ⊗ No

**Q5 What is your self-defined gender?**

- Male
- Female
- Prefer not to state

**Q6 Please rate your level of agreement (not at all to completely) with each of the following statements related to the feedback you receive from your faculty.**

- not at all agree
- slightly agree
- somewhat agree
- moderately agree
- completely agree

**Q6-1** I currently receive adequate feedback from faculty on my performance as a resident, in general.

**Q6-2** I currently receive adequate feedback from faculty on my communication skills with patients in the inpatient setting.

**Q6-3** I currently receive adequate feedback from faculty on my communication skills with patients in the clinic setting.

**Q6-4** I currently receive adequate feedback from faculty on my communication skills with my peers.

**Q6-5** I currently receive adequate feedback from faculty on my communication skills with other members of the healthcare team.

**Q6-6** I currently receive adequate feedback from faculty on my communication skills related to goals of care discussions.

**Q6-7** I currently receive adequate feedback from faculty on my professionalism skills.

**Q7 Please rate your level of agreement (not at all to completely) with each of the following statements related to the quality of the feedback you receive from your faculty.**

- not at all agree
- slightly agree
- somewhat agree
- moderately agree
- completely agree

**Q7-1** The feedback I receive from faculty is useful.

**Q7-2** The faculty that evaluate me are well-trained in providing feedback.

**Q7-3** When I receive feedback from faculty, I am usually asked to reflect on my own performance.

**Q7-4** When I receive feedback from faculty, I am usually asked to set personal goals for improvement.

**Q7-5** The written evaluations from faculty match the verbal feedback I receive.

**Q7-6** I think a coach can be helpful in providing me with real-time, useful feedback.

**Q7-7** I receive adequate feedback from resident peers.

**Q7-8** I receive adequate feedback from patients and families.

**Q7-9** I receive adequate feedback from other members of the healthcare team.

**Q7-10** I receive an adequate number of written evaluations from faculty.

**Q8 For the following statements, please rate your level of confidence (not at all to completely) about your skills.**

- not at all confident
- slightly confident
- somewhat confident
- moderately confident
- completely confident

**Q8-1** I feel confident in my ability to recognize my own strengths and weaknesses as a physician.

**Q8-2** I feel confident in setting my own goals for improvement.

**Q8-3** I feel confident in my skills as a resident in general.

**Q8-4** I feel confident in my communication skills with patients in the inpatient setting.

**Q8-5** I feel confident in my communication skills with patients in the clinic setting.

**Q8-6** I feel confident in my communication skills with my resident peers.

**Q8-7** I feel confident in my communication skills with other members of the healthcare team.

**Q8-8** I feel confident in my communication skills regarding discussion of goals of care with patients and their families.

**Q8-9** I feel confident in my professionalism.

**Q9 Please rate your level of agreement (not at all to completely) with each of the following statements.**

- not at all agree
- slightly agree
- somewhat agree
- moderately agree
- completely agree

**Q9-1** I feel supported by the residency program.

**Q9-2** I feel connected to the residency program.

**Q9-3** I feel supported by the faculty.

**Q9-4** I feel connected to the faculty.

**Q9-5** I feel supported by SUH/LPCH.

**Q9-6** I feel connected to SUH/LPCH.

**Q10** What about the Coaching Initiative excites you the most? Please describe the top 2-3 things.

**Q11** What about the Coaching Initiative concerns you the most? Please describe the top 2-3 things.

Follow-Up Survey

We are now two years into the Coaching Program and would like to understand your experiences with the program. This survey will take approximately 10 minutes and will be used to improve the Coaching program.

We truly appreciate your time and feedback. As an appreciation for your participation, twenty survey respondents will be randomly selected to each receive a $25 Amazon gift card.

Your participation in this survey is completely voluntary. All information obtained in this study is strictly confidential unless disclosure is required by law. We will not release information that could identify you, and when the results of this study are presented at a professional conference or published in a scholarly journal, the results will only be reported in aggregate, and your identity will not be divulged. All computerized data collected will be secured and password protected. Stanford University determined this study be exempt from IRB review.

Of note, you will be asked to provide your street and 2 last digits of your cell phone as personal identifiers – it will be used to link the data from the pre-coaching survey to assess for program effectiveness and to better understand potential barriers. Your identity will remain anonymous to the Coaching Program leadership.

 Please complete the survey by DATE.

Thank you from the Coaching Initiative Steering Group.

**Q1 Please answer the following questions to de-identify your survey responses:**

- Street you lived on as a child
- Last 2 digits of cell phone

**Q2 What postgraduate year (PGY) did you just complete?**

- 1
- 2
- 3
- 4
- 5

**Q3 Which residency program are you in?**

- Neurology Adult
- Neurology Pediatric
- Surgery General
- Surgery Plastics
- Surgery Vascular

**Q4 What is your self-defined gender?**

- Male
- Female
- Non-binary
- Prefer not to state
- Other

**Q5 What is most typical setting of your observed encounters with patients?**

- Inpatient
- Resident Continuity Clinic
- Outpatient Elective
- Clinic Block
- Other, please describe

**Q6 How does your coach typically observe your encounter with patients?**

- In person
- Could see and hear the encounter
- Could only hear the encounter
- Other, please describe

**Q7 How do you typically receive feedback from your coach?**

- In person
- Video call
- Phone call
- Email
- Other, please describe

**Q8 Please rate your level of agreement (not at all to completely) with each of the following statements related to the feedback you receive from your faculty coach.**

- not at all agree
- slightly agree
- somewhat agree
- moderately agree
- completely agree

**Q8-1** I currently receive adequate feedback from my faculty coach on my performance as a resident, in general.

**Q8-2** I currently receive adequate feedback from my faculty coach on my communication skills with patients in the inpatient setting.

**Q8-3** I currently receive adequate feedback from my faculty coach on my communication skills with patients in the clinic setting.

**Q8-4** I currently receive adequate feedback from my faculty coach on my communication skills with my peers.

**Q8-5** I currently receive adequate feedback from my faculty coach on my communication skills with other members of the healthcare team.

**Q8-6** I currently receive adequate feedback from my faculty coach on my communication skills related to goals of care discussions.

**Q8-7** I currently receive adequate feedback from my faculty coach on my professionalism skills.

**Q9 Please rate your level of agreement (not at all to completely) with each of the following statements related to the quality of the feedback you receive from your faculty coach.**

- not at all agree
- slightly agree
- somewhat agree
- moderately agree
- completely agree

**Q9-1** My faculty coach has supported my wellbeing as a resident.

**Q9-2** The feedback I receive from my faculty coach is useful.

**Q9-3** My faculty coach is well-trained in providing feedback.

**Q9-4** When I have a session with my faculty coach, I am usually asked to reflect on my own performance.

**Q9-5** When I have a session with my faculty coach, I am usually asked to set personal goals for improvement.

**Q9-6** I receive adequate feedback from resident peers.

**Q9-7** I receive adequate feedback from patients and families.

**Q9-8** I receive adequate feedback from faculty members who are not my faculty coach.

**Q10 For the following statements, please rate your level of confidence (not at all to completely) about your skills.**

- not at all confident
- slightly confident
- somewhat confident
- moderately confident
- completely confident

**Q10-1** I feel confident in my ability to recognize my own strengths and weaknesses as a physician.

**Q10-2** I feel confident in setting my own goals for improvement.

**Q10-3** I feel confident in my skills as a resident in general.

**Q10-4** I feel confident in my communication skills with patients in the inpatient setting.

**Q10-5** I feel confident in my communication skills with patients in the clinic setting.

**Q10-6** I feel confident in my communication skills with my resident peers.

**Q8-7** I feel confident in my communication skills with other members of the healthcare team.

**Q10-8** I feel confident in my communication skills regarding discussion of goals of care with patients and their families.

**Q10-9** I feel confident in my professionalism.

**Q11** What about the Coaching Program has been helpful to you?

**Q12** What about the Coaching Program has not been helpful to you?

**Q13** What we can do to improve the Coaching Program so it better meets your needs?

**Q14** Any additional comments/thoughts about the Coaching Program?
