# Supplementary material for: Impact of a coaching program on resident perceptions of communication confidence and feedback quality
Source: BMC Med Educ. 2024 Apr 22;24:435. doi: 10.1186/s12909-024-05383-5 (PMC11036561; doi:10.1186/s12909-024-05383-5)
Supplement: Supplementary file 2 — Supplementary Material 2. [file 12909_2024_5383_MOESM2_ESM.docx]

**Additional File 2:**

Supplemental Data for Sensitivity Analysis

Unpaired analysis of *all* baseline survey responses compared to *all* follow-up survey responses:

| **Domain** | **Question/Statement** | **Baseline**  **Survey N=126** | **Follow-Up**  **Survey**  **N=46** | **p-value** |
| --- | --- | --- | --- | --- |
| Communication with **patients** | *I feel confident in my communication skills:* | | | |
|  | With patients in the inpatient setting. | 3.8 | 4.2 | **0.004** |
|  | With patients in the clinic setting. | 3.7 | 4.1 | **0.003** |
|  | Regarding goals of care discussions with patients and their families. | 3.3 | 3.9 | **<0.001** |
| Communication with **others** | *I feel confident in my communication skills:* | | | |
|  | With resident peers. | 3.9 | 4.2 | **0.02** |
|  | With other members of the healthcare team. | 3.9 | 4.2 | **0.01** |
| Self-reflection and goal setting | *I feel confident in:* | | | |
|  | My skills as a resident in general. | 3.3 | 3.7 | **0.01** |
|  | My ability to recognize my own strengths and weaknesses as a physician. | 3.4 | 4.2 | **<0.001** |
|  | Setting my own goals for improvement. | 3.6 | 4.2 | **<0.001** |

| **Question/Statement** | **Baseline**  **Survey**  **N=126** | **Follow-Up**  **Survey**  **N=46** | **p-value** |
| --- | --- | --- | --- |
| *I currently receive adequate feedback from faculty [my faculty coach] on:* | | | |
| My performance as a resident, in general. | 3.3 | 3.8 | **0.01** |
| My communication skills with patient in the inpatient setting. | 2.8 | 3.3 | **0.03** |
| My communication skills with patients in the clinic setting. | 2.6 | 3.8 | **<0.001** |
| My communication skills with my peers. | 2.3 | 2.8 | **0.04** |
| My communication skills with other members of the health care team. | 2.7 | 2.9 | 0.34 |
| My communication skills related to goals of care discussions. | 2.8 | 2.9 | 0.56 |
| My professionalism skills. | 3.2 | 3.7 | 0.05 |
| *I receive adequate feedback from faculty members [who are not my faculty coach].* | 3.0 | 3.7 | **<0.001** |
| *The feedback I receive from faculty [my faculty coach] is useful.* | 3.5 | 4.2 | **<0.001** |
| *The faculty [my faculty coach] are [is] well-trained in providing feedback.* | 3.0 | 4.3 | **<0.001** |
| *When I receive feedback from faculty [my faculty coach] I am usually asked:* | | | |
| To reflect on my own performance. | 3.5 | 4.6 | **<0.001** |
| To set personal goals for improvement. | 3.1 | 4.3 | **<0.001** |
